# The impact of military activities on the concentration of mercury in soils of military training grounds and marine sediments

**DOI:** 10.1007/s11356-016-7436-0

**Published:** 2016-09-03

**Authors:** Karolina Gębka, Jacek Bełdowski, Magdalena Bełdowska

**Affiliations:** 1 The Institute of Oceanography, University of Gdansk, Marszałka Piłsudskiego Alley 46, 81-378 Gdynia, Poland; 2 The Institute of Oceanology, Polish Academy of Sciences, Powstańców Warszawy Street 55, 81-712 Sopot, Poland

**Keywords:** Mercury, Ammunition, Sediment, Soil, Military training ground, Gun range

## Abstract

Military activities have been conducted on land and at sea. Both during conflicts and in peace time, some regions served as a military training ground which included firing positions and bunkers. Mercury fulminate has been used in ammunition primers and detonators. Certain amount of ammunition was dumped into the Baltic Sea after the Second World War. Because of corroded containers, mercury can be released into the marine environment. The soil and sediment samples were taken from military training grounds, southern Baltic in 2014 and 2015. The concentration of mercury was determined by AMA-254 analyzer. Hg concentration was higher in the places of military activities, as compared to other areas. Ten times increased concentration of Hg was determined in soil sample collected in area of active gun range compared to the reference station. The significant higher concentration of mercury was detected in stations where chemical warfare agents were found.

## Introduction

Heavy metals still remain a serious factor threatening natural environment. Mercury is considered the most dangerous of them (Polak-Juszczak 2009). It poses a threat to humans even at low concentration. Toxicity of mercury depends on the form it occurs in. Methylmercury is the most harmful form of mercury and is formed in a process of methylation of ionic mercury (Boeing 2000). The diagenetic processes which He undergoes in marine environment are still being investigated worldwide. The reason for this is numerous cases of poisoning, often lethal. Such events occurred in a Japanese fisherman village, Minimata. The residents died after consumption of fish which contained the amount of mercury above 100 μg/g (Duffy and vanLoon 2008). Thus, consumption of fish is for mercury one of dominant ways to enter a human organism. Moreover, an increase in the number of people with brain damage, who live in the mercury contaminated areas, has been observed for several years which was attributed to the increase of Hg concentration in the environment (Bernard et al. 2001). Mercury can enter a human body also through the respiratory system and skin. He has a negative impact on the kidneys, lungs, digestive system and skin (Bose-O’Reilly et al. 2010). Research has shown that mercury has the biggest impact on organisms, which have not yet shaped their resistance. Children are the most vulnerable to hazards occurring in the environment, in particular exposure to physical and chemical factors that can lead to negative health effects before achieving full maturity. Mercury damages nerve cells which in turn, prevents normal development of the central nervous system (Zahir et al. 2005).

Heavy metals can accumulate and translocate in soil environment which causes deterioration of the environment (Chang et al. 2014). Process of methylation occurs both in marine and terrestrial environment. Concentration of metals in vegetables and fruits is higher than in soils due to accumulation in edible parts of these crops. Uptake of heavy metals by crops depends on several factors, such as plant species, growth stage, and soil type (Orisakwe et al. 2012). Not only typical farmed vegetables contain the Hg but also other plants like mushrooms, which occur in natural environment and represent a group of bio-indicators. The mushrooms accumulate from the subsoil’s most toxic form of mercury, methylmercury (Kalač et al. 1996). Some of which are edible for humans (Kalač and Svoboda 2000). Vegetables can absorb metal in amounts which can lead to human health problems (Orisakwe et al. 2012). Mercury in soils can be reduced to Hg^0^ by emission to the atmosphere (Siemiński 2007). Emission of mercury from the forest soils to the air is dependent on plenty of factors such as solar radiation, temperature, and soil moisture (Choi and Holsen 2009; Kuiken et al. 2008). Within 24 h of deposition, 0.1 to 3.0 % Hg is emitted back to the atmosphere (Ericksen et al. 2005; Hintelmann et al. 2002).

Sediments are a major sink of mercury in marine environment which represent a dynamic environment where pollution is retained and undergo a process of diagenesis, which leads to chemical transformations (Seisuma and Kulikowa 2012). Transformation of mercury in sediments, bioavailability, and remobilization of Hg depend on mercury speciation. Both the transportation and form of Hg are important for its distribution in sediments (Bełdowski and Pempkowiak 2007). Mercury methylation occurs in marine environment, and as an ion of methylmercury, it is the most bio-available form and bio-accumulates easily in organisms (e.g., in predatory fish). This process occurs mainly in sediments. Moreover, Hg is readily transported in marine environment (Szymalska et al. 2010). As a result of this process, Hg enters to the benthic organisms and then into the seafood and fish which are consumed by human.

In the past, mercury was used in the production of measuring devices such as thermometers, barometers, manometers etc. Moreover, He was an important ingredient used to manufacture ammunition primers. Military activities with the use of mercury were conducted on land since 1850’s, when caplocks were invented. Different types of guns and ammunitions were used there. This has left their marks on soil. Training grounds and gun ranges are specific areas where guns are used regardless of the state of war. Soldiers and civilian practice there as well. These kinds of places are often forested, and the surrounding area is available for other people, who collect mushrooms and forest fruits.

After WW II (World War II), considerable amounts of captured German chemical ammunition were dumped into the Baltic Sea, including mainly aerial bombs, artillery shells, and grenades (Ostojski et al. 2010). Depending on susceptibility to initiation, explosives can be divided into three groups: primary, secondary, and tertiary ones. Primary explosives include silver azide, lead styphnate, and mercury fulminate. Because of these components, materials are extremely sensitive to initiation. Primary explosives are used to initiate the next group, secondary explosives, to “firing train” (Pichtel 2012). In chemical munitions, explosives were used as propellants (in artillery shells and mortar grenades) and as bursting charges. Some of those explosives used mercury fulminate as primers (HELCOM 2013). The official dumpsites are located in the east of Bornholm and southeast of Gotland. Research conducted in the framework of CHEMSEA project showed that munitions were dumped also in unofficial sites such as Gdansk Deep and Slupsk Furrow (Bełdowski et al. 2014). Factors such as mechanical erosion, contact with sea water, biotic and abiotic processes, environmental conditions, etc. have a deep impact on corrosion of munitions and degradation of its constituents. Depending on type of ammunition, thickness of material it was made of, the ammunition starts to corrode (Korzeniewski 1994; Bełdowski et al. 2014). The ammunition can be a new potential source of Hg in the Baltic Sea because of the usage of mercury in primers—as mercury fulminate (Della Torre et al. 2010; Pichtel 2012). Mercury can remobilize from sediments causing a further threat to marine organisms. The largest effects of mercury presence in the sediments and water near the bottom are visible in the benthic organisms (Polak-Juszczak 2012). Recent studies have confirmed that mercury concentration in fish inhabiting the dumpsites (*Conger conger* 1.53 ± 0.13 [mg/kg w.w.]; *Helicolenus dactylopterus* 2.23 ± 0.22 [mg/kg w.w.]) is much higher than in the same species living in the unpolluted environment (*C*. *conger* 0.67 ± 0.18 [mg/kg w.w.]; *Helicolenus dactylopterus* 0.63 ± 0.04 [mg/kg w.w.]) (Della Torre et al. 2010).

HELCOM Commission has introduced an action plan connected with reduction of the emission of Hg to environment because of mercury toxic properties, bearing in mind that human activity has the biggest impact on Hg introduction to the environment. Helsinki Commission has implemented a plan connected with the reduction of heavy metals use in architecture materials (HELCOM 2011). By a decision from 2009, it is forbidden to sell mercury in thermometers and other measuring devices used for general public.

The introduction of this restriction caused the reduction of Hg in the Baltic States. The annual deposition of mercury in 1990–2010 was reduced to about 35 % (Bartnicki et al. 2013). Even though the supply of mercury has been reduced, concentration of it in the Baltic Sea still remains high. It can be the result of local anthropogenic sources like dumped ammunition. The aim of the present work was to determine the effect of military activities both on soils of military trainings grounds and marine sediments.

## Materials and methods

**Fig. 1:**
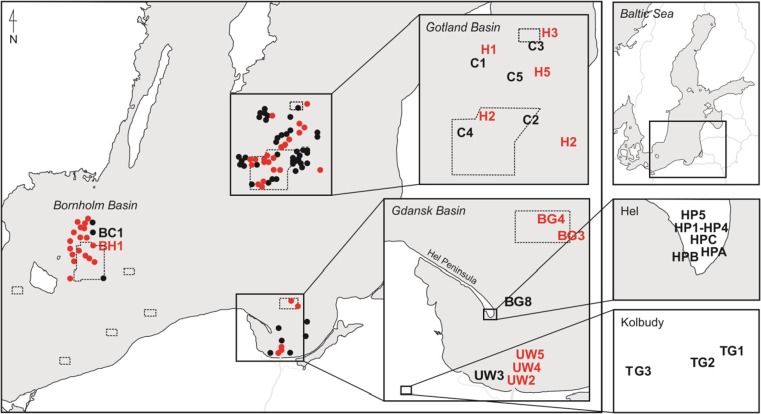
Sampling location (*red color*—marks of CWA; *black color*—no marks of CWA; the *dotted line*—dumping zones for chemical munitions) (Bełdowski et al. 2014)

Soil samples were collected in years 2014 and 2015. Some samples were collected from inactive military training grounds. One of them is Hel Peninsula, which was the territory of the major military base. Firing positions and bunkers are still present there, but the area is no longer active. Samples from Hel Peninsula were taken from eight points. The first point was located around old no. 2 firing position (HPB), the second one next to no. 3 firing position (HPA), and the last one was located around former backup fire control point fire (HPC). The samples were taken from 5 and 20 cm depth. Next five stations were located in different parts of Hel Peninsula. The first one was located on the firing line (70 m) (HP1), the second one about 1 m behind the front firing line (HP2), and the next one about 1 m in front of the firing line (HP3). Point no. 4 was located on firing line (50 m) (HP4) and the last one in reference area (HP5). Samples were taken from 5, 20, and 30 cm depth from every station. Soil samples were collected also from military inactive training grounds which was lactated in Kolbudy (TG1). Kolbudy is a small town about 20 km from Gdansk (Fig. 1). Drills connected with using heavy guns used to take place there quite often (http://historia.trojmiasto.pl/Poligony-opuszczone-przez-wojsko-zabudowane-osiedlami-n40032.html). Currently, this ground is military inactive, but it is facilitated to private use. Soil samples were taken from 5, 20 and 30 cm. Moreover, soil samples were taken from reference area located about 50 m (TG2) and 1500 m from active training ground (TG3). Soil samples were taken from 5, 20, and 30 cm depth. Thirty soil samples were collected altogether (21 from Hel Peninsula and nine from Kolbudy). After that, samples were put into the string bags and frozen.

Marine samples were collected during cruises of s/y. The samples from Gotland Basin (97 samples) and Bornholm Basin (21 samples) were taken in the framework of CHEMSEA project in 2012–2013. Samples from Gdansk Basin (eight samples from Gdansk Deep and five samples from Vistula Mouth) were taken in the framework of MODUM project in 2014 (Fig. 1). The stations where leaking and chemical munitions were detected and were confirmed on the basis of detection of clark, adamsite, and mustard gas degradation products. For the estimation of ammunition effects, mercury concentrations in sediments from these stations were compared to stations located ca. 100 m away (chemical warfare agents (CWA) concentration below detection level).

Samples located near the dumped munitions were collected by the underwater vehicle (ROV Super Achille) at the distance of approx. 0.5 m from corroded munitions. The rest of the samples were collected by a box-corer. After sampling, the samples were put into the string bags and frozen.

### Chemical analyses of Hg and other parameters

All samples were preserved at −12 °C until the time of chemical analysis. The samples had been homogenized and freeze-dried before analyzing. The concentration of mercury was determined by AMA-254. Recovery of the methods amounted to 98 %, whereas standard deviation was below 5 %. The limit of quantification was 0.01 ng/g.

Additionally, a grain size analysis was performed by sieve analysis. This analysis was not use for samples from Bornholm Basin and Gotland Basin. The content of organic matter was obtained by loss of ignition in 550 °C (Ciborowski 2010).

## Results

### Land samples

**Fig. 2:**
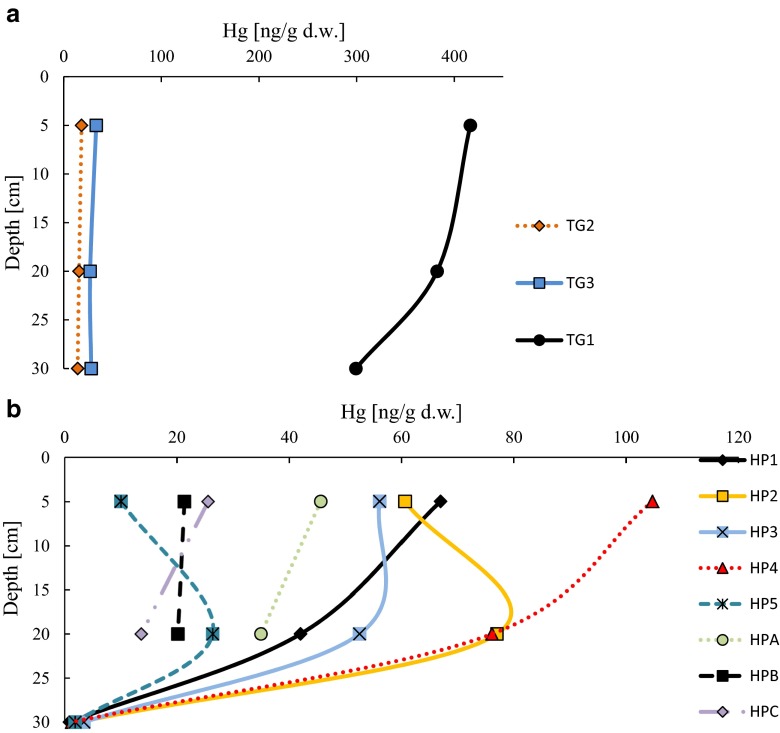
Concentration of Hg [ng/g d.w.] on 5, 20, and 3 cm layers of trainings ground soils of Kolbudy (**a**) and layers of Hel Peninsula soils (**b**) (the range of scale on **a** figure is different than on **b** figure)

Kolbudy is a village located about 20 km from Gdańsk (northern part of Poland), with population of ca. 3500. No large scale industrial activities are present there. There are many residential buildings and one inactive training ground. This training ground was active till the 1990s. Nowadays, this area has few hectares of forest. Some part of this inactive training ground is occupied by gun range. Due to forested area, organic matter concentration varied from 2.3 to 5.8 % in all station from inactive training ground (Table 1). Concentration of mercury in soil samples taken from inactive training ground in Kolbudy was characterized by high variability. Similar concentration of Hg was found in the samples which were taken from the areas situated about 50 and 1500 m from the gun range. Concentration of mercury in these stations ranged from 18.3 to 33.3 ng/g in surface layer and from 14.3 to 28.1 ng/g at 30 cm depth. A significantly higher concentration of Hg was noticed in the station localized in active gun range area. Concentration of mercury in soil taken from surface layer amounted to 416.2 ng/g. Concentration decreased with depth to 299.6 ng/g (Fig. 2a).

**Table 1:** Characteristics of land stations

Description of station	Symbol of station	LOI (%)		
		Depth (cm)		
		5	20	30
Hel Peninsula-inactive gun range; line of fire (70 m)	HP1	5.15	3.98	0.29
Hel Peninsula-inactive gun range; 1 m behind line of fire	HP2	13.97	10.17	1.11
Hel Peninsula-inactive gun range; 1 m in front of line of fire	HP3	12.15	6.01	0.75
Hel Peninsula-inactive gun range; line of fire (50 cm)	HP4	7.40	5.95	0.17
Hel Peninsula-forest area	HP5	6.99	9.06	0.62
Hel Peninsula-inactive firing position no.3	HPA	7.82	6.68	–
Hel Peninsula-inactive firing position no.2	HPB	2.27	5.47	–
Hel Peninsula-inactive former backup point fire control	HPC	2.34	1.12	–
Kolbudy-inactive training ground—area of active gun range (shooting position)	TG1	5.78	3.78	3.72
Kolbudy-inactive training ground—50 m from active gun range	TG2	5.52	2.54	2.26
Kolbudy-inactive training ground—1.5 km from active gun range (forest area)	TG3	5.85	2.98	2.49

Hel Peninsula was used as a main major military base of Poland during both world wars. After Second World War, active training grounds were located there. Nowadays, Hel Peninsula is a tourist destination. Population of the town is 3700. Concentration of mercury in soil samples taken from Hel Peninsula was characterized by large variability in the surface layer. The highest concentration of mercury was recorded in HP4 station (104.6 ng/g). This station was located on the line of fire on Hel Peninsula inactive gun range. The lowest concentration of Hg was detected in forest HP5 station (10.3 ng/g). Concentration of mercury decreased with depth. Concentration of this metal was comparable for all stations in the deepest layer and it ranged from 0.6 to 1.1 ng/g (Fig. 2b). The soil samples from Hel Peninsula contained more organic matter (from 0.2 to 14.0 %) than soil samples from Kolbudy. In both cases, content of organic matter decreased with depth (Table 1).

### Marine samples

**Fig. 3:**
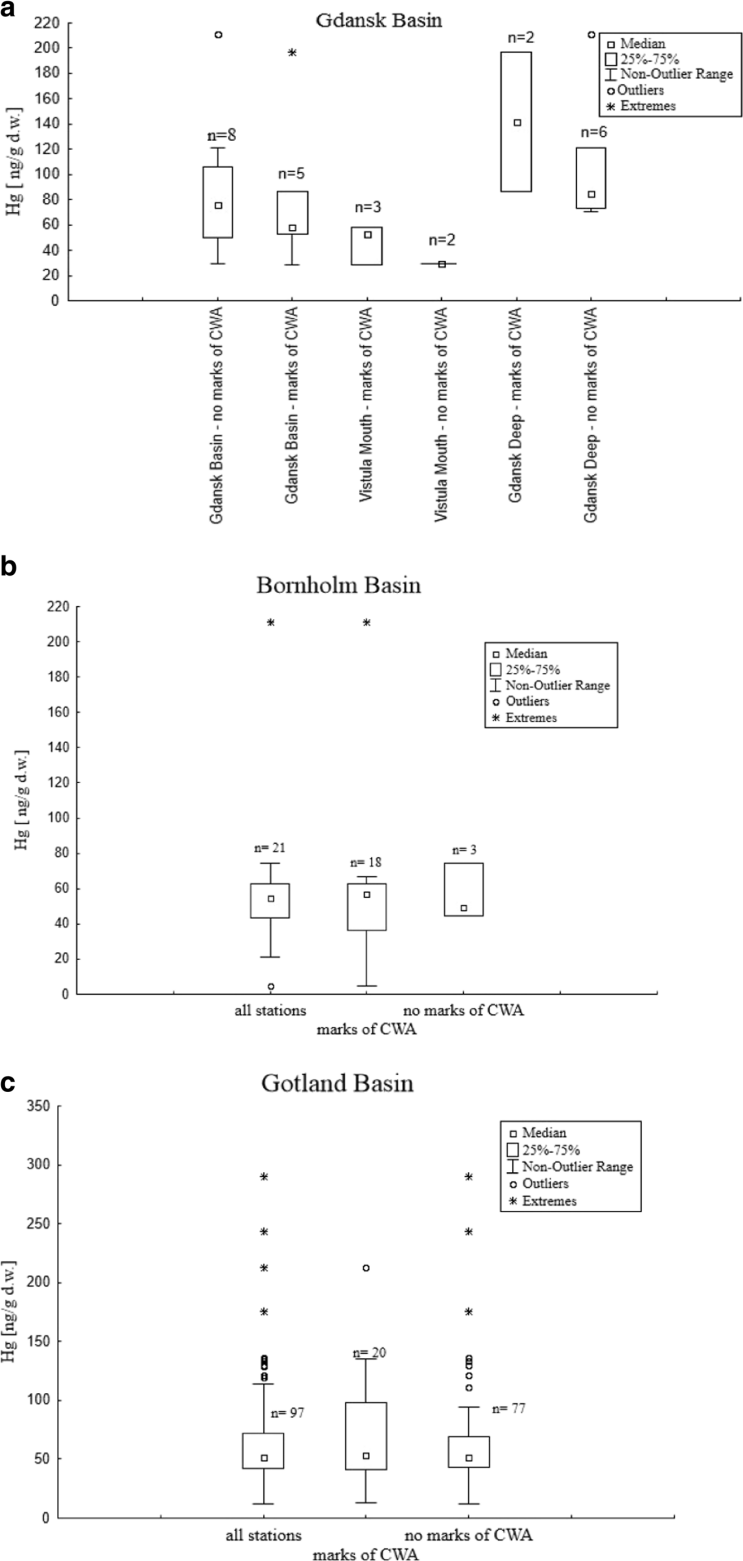
Statistical characteristics of mercury concentration [ng/g d.w.] in **a** the Gdansk Basin divided in Gdansk Deep and Vistula Mouth, **b** the Bornholm Basin, and **c** the Gotland Basin, where CWA was detected or was not detected

Concentration of mercury in all marine sediments from the Bornholm, Gotland, and Gdansk Basin varied in the range between 4.9–211.1 ng/g, 11.9–289.9 ng/g, and 3.8–211.4 ng/g, respectively (Fig. 3).

Area of Gdansk Basin was divided in two parts (Gdansk Deep and Vistula mouth). This division was based on proximity of pollutant sources and geological features such as depth and type of sediments. The highest median concentration of mercury was detected in Gdansk Deep sediments, where CWA degradation products were found (142.3 ng/g). Nonetheless, taking into account all the stations located in the Gdansk Basin, median of concentration of Hg in stations with CWA was 59.8 ng/g. The non-outlier range of concentration of mercury in the Gdansk Basin sediments ranged from 3.8 to 121.1 ng/g. Additionally, extreme concentration of mercury was detected in one station where dumped ammunition was found (196.4 ng/g) (Fig. 3a).

The median of concentration of Hg in Bornholm Basin sediments was 54.8 ng/g. However, the median of concentration of mercury, in stations where chemical warfare agent degradation products were detected, was 56.8 ng/g. The non-outlier range in all stations located in the Bornholm Basin ranged from 20.9 to 74.6 ng/g. The extreme concentration of mercury was detected in one of the stations, where CWA was found (211.1 ng/g) (Fig. 3b).

The median of concentration of Hg in Gotland Basin stations where CWA was detected amounted to 53.0 ng/g, whereas the median for sediments in the whole area of the Gotland Basin amounted to 51.7 ng/g. The non-outlier range for all stations ranged from 11.9 to 114.1 ng/g. Nonetheless, the extreme concentration of Hg was found in the station where CWA was not detected (289.9 ng/g) (Fig. 3c).

## Discussion

### Land samples

**Fig. 4:**
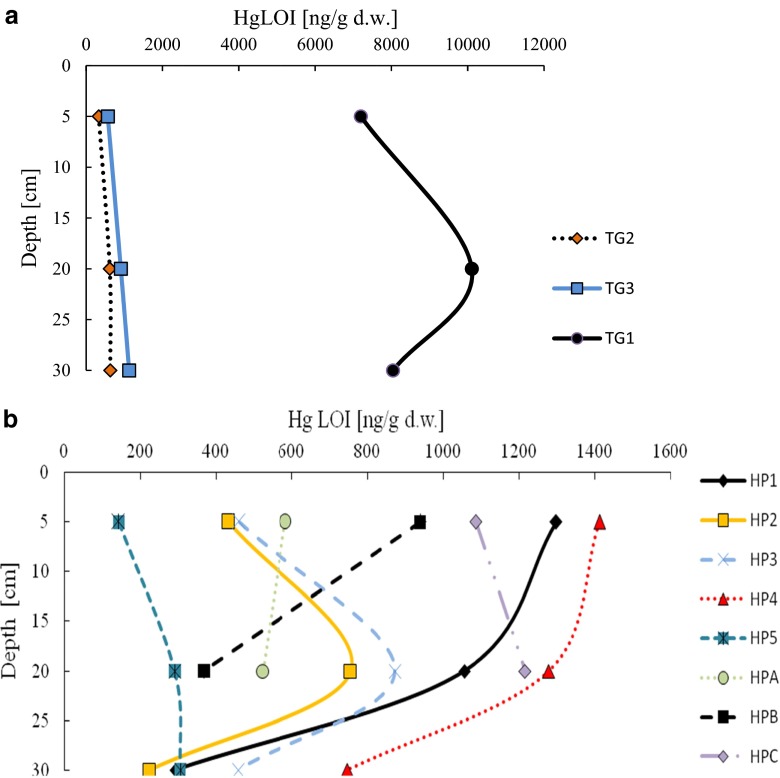
Concentration of Hg normalized to content of organic matter (Hg_LOI_) on 5, 20, and 30 cm layers of trainings ground soils of Kolbudy (**a**) and layers of Hel Peninsula soils (**b**)

Important sources of Hg in environment are wet and dry atmospheric deposition (Radke and Piketh 2013). The terrestrial plants absorb mercury mostly from soil solution by the root system. (Bondada et al. 2004). As a result of decay of the plants, absorbed mercury can be release back to the soil and leads up to enrichment of soil in Hg which becomes more available to plants and more mobile than metal occurring in soil solution (Perronnet et al. 2000). Usage of firearms can contribute to additional enrichment of land in mercury—especially soil containing significant amount of organic matter and fine grains. Usage of firearms which ammunition contain mercury compounds becomes a new source of Hg in areas where it is used. Places such as areas of military activities or gun ranges were characterized by increased concentration of mercury. It is especially important in areas where wild game lives and where forest fruits and mushrooms are collected by humans. These activities are very popular in European countries like Poland. Changes in concentration of mercury in soil samples can be a result of the differences in organic matter and fine fraction content. To enable comparison, concentration of Hg was normalized to fine fraction and organic matter content. Due to the small variability of fine grain contribution in soil samples, only results of concentration of mercury on organic matter (Hg_LOI_) are presented. The highest concentration of Hg_LOI_ was detected in the 20 cm layer at the station located in inactive training grounds where gun range is presently in operation (10,108.2 ng/g) (Fig. 4a). Differences between concentrations of Hg_LOI_ in various layers can be a result of mixing of the soil during scouring. Small enrichment in organic matter was detected in the station located from 50 to 1500 m from gun range. Significant differences between Hg_TOT_ and Hg_LOI_ in stations localized in and out of gun range area (TG1-TG3) can be a result of firearms usage. Concentration of mercury in TG1 was ten times higher compared to reference areas such as TG2 and TG3 stations. It means that military activity contributed to the Hg concentration. It is in particular noticeable in the surface layer of soil. A lower concentration of mercury occurred on surface and deeper layers in TG2 and TG3 stations (Fig. 4a). It showed significant impact of using ammunition on contamination of soil by Hg.

Concentrations of mercury normalized on the content of organic matter (Hg_LOI_) in samples taken from the Hel Peninsula were characterized by high variability. The most enriched in mercury organic matter was observed in HP4 station (1413.3 ng/g). However, concentration of Hg_LOI_ in this station was seven times lower than Hg_LOI_ in TG1 station. The lowest concentration of Hg_LOI_ was observed in the HP5 station located in forest area (143.6 ng/g). The downward trend with depth was observed in all the stations. The range of concentration of Hg_LOI_ in the deepest layer (30 cm) amounted from 225.0 to 747.2 ng/g (Fig. 4b).

A much higher concentration of Hg, compared to Hel Peninsula soil, was detected in urban soil of town garden in Wexford (680 ng/g) (McGrath 1995). The highest concentration of Hg which was found in the soil samples taken from active gun range (but inactive training ground) was similar to the highest concentration of this metal in urban soils in Beijing (260 ng/g) (Chen et al. 2010), where mercury is delivered mostly by atmospheric deposition from industry. Mean concentration of mercury detected in reference area (HP5 station) located in Hel Peninsula soil (13 ng/g) was lower than concentration of Hg which was found in Hel Peninsula by Falandysz et al. (2012) (40 ng/g). Moreover, concentration of mercury from active gun range (Kolbudy—area of inactive military training ground) was similar compared to mean concentration of Hg in unsorted waste in the municipal landfill in Szadółki (309 ng/g). High concentration of mercury occurs in biodegradable waste in the landfill as well (564.6 ng/g—area of old waste disposal site in Gdansk Letnica). This is caused by absorption properties of plants (Falkowska et al. 2013).

### Marine samples

Mercury emission estimation from Baltic Sea countries have decreased in period from 1990 to 2011 by about 74 % (HELCOM 2013). So-called inhalation levels of concentration of mercury are not a threat for humans. Exceptions are huge industrial sources, technological failures, and ecological disasters. The most dangerous are historical deposits of mercury accumulated in marine sediments and soils. Due to high temperature, mercury from sediments and soils can disrupt the balance circulation of Hg in environment and then contribute to increase of concentration of this metal in food chain.

Occurrence of elevated Hg levels in sediments in individual regions of the Baltic Sea depends on the localization of the sampling station (Bełdowski et al. 2014). It can be connected with the distance from the coastline or the mouth of the rivers but also with the content of fine grains and organic matters (Pempkowiak 1997). There are five main accumulation mechanisms of trace elements in or on sediments: adsorption of fine grains materials, co-precipitation with hydrous oxides of Fe and Mn, precipitation of metal compounds trace, adsorption and complexation by organic matters, and incorporation into the crystalline minerals (Pempkowiak 1997).

Higher concentration of mercury could be also connected with weapons deposited in the sediments. This is related to using mercury to produce detonators in bombs and shells, which were dumped into the sea (Della Torre et al. 2010). A lot of factors, such as type of chemical, contact with sea water, mechanic erosion, and type of ammunition dumped have an impact on this. Due to the dynamic processes (i.e., currents, sediment oxidation, and mixing), containers corrosion rates depend on the thickness of the walls and environmental conditions (Bełdowski et al. 2014; Korzeniewski 1998). It has been showed that ammunition which are buried in silty sediments are not rapidly degraded. Thin-crust bombs (thickness 1.5–3.0 mm) which occur on hard bottom are the most degraded (Korzeniewski 1998).

The Gdansk Basin is characterized by clay and silk sediments. Distribution and diversity of the bottom sediments are connected with the depth of the Baltic Sea and the shape of shoreline. In the shallow zone, hydrodynamic processes prevent stable deposition of fine grain sediments. Hence, in this type of area, sands and gravelly sands dominate (Uścinowicz 2011).

**Fig. 5:**
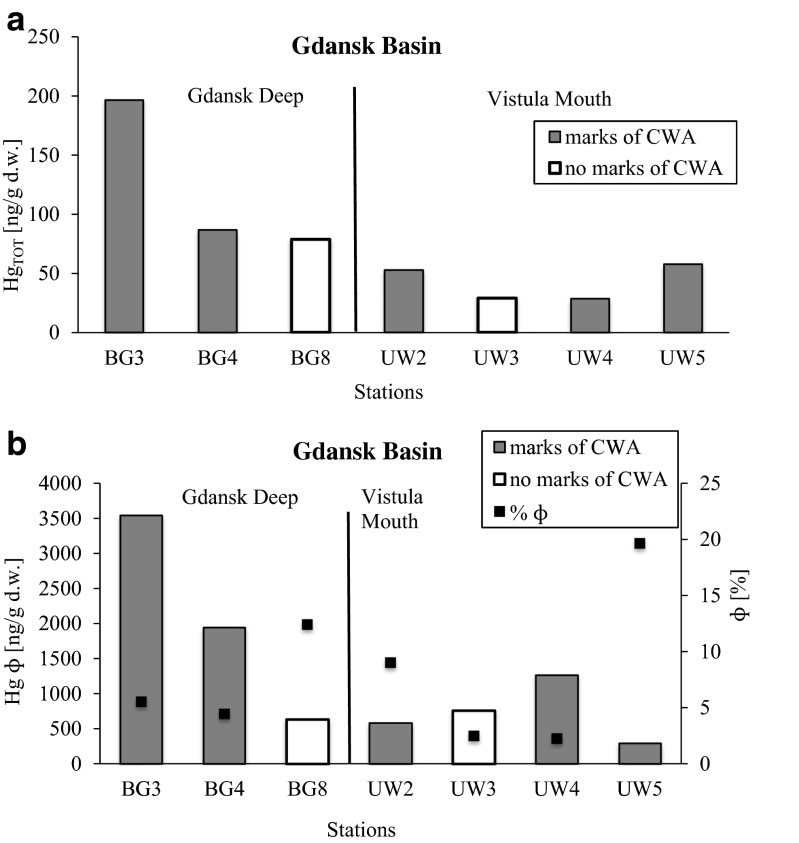
Concentration of Hg [ng/g d.w] (**a**) and concentration of Hg normalized to content of fine grains (Hgɸ) in individual stations of Gdansk Basin where CWA was detected or CWA was not detected (**b**)

CWA was detected in five out of 15 analyzed sediments located in the Gdansk Basin. The highest concentration of Hg in the Gdansk Basin region was found in accumulation area: Gdansk Deep sediments. The content of fine grain in Gdańsk Basin sediments was from 2.6 to 19.7 % (Table 2). Gdansk Deep is a location where pollutants accumulate. The content of fine grains in an increase of Hg concentration of about 149 % compared to the reference station (BG8) was observed in BG3 station (100 m of depth). The lowest increase of Hg concentration (10 %) was observed in BG4 station localized in Gdansk Deep (99 m of depth) (Fig. 5a). Due to the high impact of the fine fraction on Hg concentration in sediments, the results of investigation were normalized (Hgɸ) (Table 2). After taking fines into account, a fourfold increase of Hgɸ was observed in BG3 station compared to the reference BG8 station. Significantly enriched in mercury were sediments from BG4 station as well (Fig. 5b). It indicates that enriched fine grains were detected in ammunition dumpsites. It confirms the hypothesis that dumped ammunition probably has a significant impact on concentration of Hg in Baltic Sea sediments.

**Table 2:** Characteristics of individual stations of the Gdansk Basin

Description of station		Symbol of station	ϕ (%)
Area of Gdansk Deep	Marks of CWA	BG3	5.54
	Marks of CWA	BG4	4.46
	No marks of CWA	BG8	12.43
Area of Vistula Mouth	Marks of CWA	UW2	9.04
	No marks of CWA	UW3	2.50
	Marks of CWA	UW4	2.26
	Marks of CWA	UW5	19.67

Despite the fact that Gdansk Deep is a deposition area where pollutants are transferred, CWA were also identified in the area of the mouth of the Vistula. Nonetheless, the area was scanned by the multibeam echosounder (MBES) and side scan sonar (SSS), and it showed that in the bottom of that area ammunition was not found (Bełdowski et al. 2014). CWA, which was detected in area of Vistula mouth, can be the result of the transfer of the contents of chemical weapons, including mercury from unmarked areas of dumped ammunition, i.e., by fishing vessels. Moreover, the fine fraction from the station UW4, where a CWA was detected, proved to be more enriched in mercury than reference UW3 station (about 44 %). Concentration of mercury in UW2 and UW5 stations was higher by about 81 and 99 % respectively, compared to the reference area (Fig. 5b). However, slightly enriched fine grain in mercury in UW2 and UW5 station was recorded. Taking into account the composition of the sediment grain size, increase of Hg in this area is connected with what is contained in fine grains. The cause might have been due to Vistula mouth being an area of transport and not pollution accumulation.

**Fig. 6:**
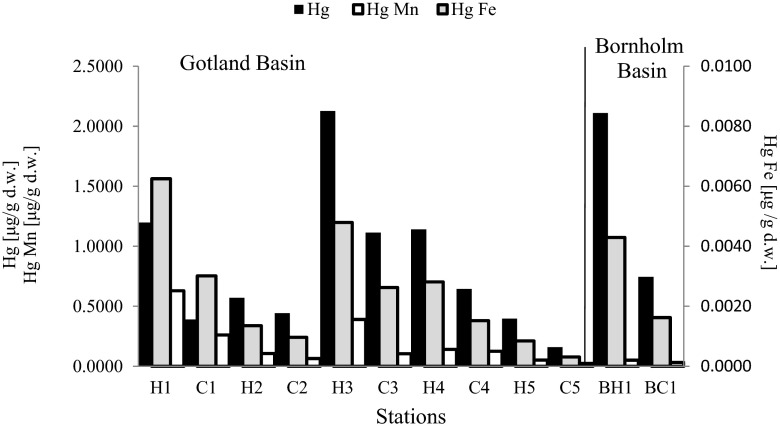
Concentration of Hg [μg/g d.w.] and concentration of Hg normalized on concentration of Mn and Fe [μg/g d.w.] in individual stations of the Bornholm Basin and Gotland Basin (*H* marks of CWA, *C* no marks of CWA (reference area))

CWA was detected in 18 out 21 stations localized in Bornholm Basin. Significant increase of the concentration of Hg was found in the station BH1 where mustard gas was detected. Concentration of Hg in this station was about 183 % higher compared to the reference area (BC1) (Fig. 6). Both of these stations were characterized by sandy and clay sediments. What is more, concentration of Fe and Mn in BH1 station was higher than in BC1 station. The rest of the stations were characterized by lower concentration of Hg. It indicates that conventional ammunition which was dumped into Bornholm Basin area has not corroded yet.

CWA was detected in 20 out 77 analyzed stations situated in the Gotland Basin. A likely leakage of Hg, which probably comes from dumped munitions, was detected in five stations of the Gotland Deep. The greatest increase was found in H1 station, where concentration of Hg increased by about 206 % compared to the reference area (C1). An increase by 149 % was found in H5 station compared to reference area (C5). Both stations (H5 and C5) were located at 110 m of depth. A considerable increase of Hg concentration was detected in H3 station (91 %) compared to the C3 station. The lowest impact (29 %) of leaking mercury was detected in the central part of the Gotland Deep (Fig. 6). Nevertheless, not all stations with detected CWA were characterized with higher mercury concentration. Correlation between concentration of mercury and concentration of CWA in the Baltic Sea sediments was not found. However, increase of concentration of Mn and Fe was detected in ammunition dumpsites compared to reference area (Fig. 6).

The dumped ammunition was detected in the Irish Sea too. However, concentration of Hg was lower than in Baltic Sea dumpsites (50 ng/g) (Callaway et al. 2011). Among other individual regions of the Baltic Sea, concentration of mercury in the Gdansk Basin, Bornholm Basin, and Gotland Basin sediment was not high. Significantly higher concentration of Hg was noticed in Gulf of Bothnia (270 ng/g) (Leivuori et al. 2000), where historical pollution of cellulose plants introduced large quantities of this metal. A much higher concentration of mercury was found in the Bay of Mecklenburg, where a mean value of 180 ng/g was detected (Leipe et al. 2005)—there, Hg source was probably chemical plant in Schkopau, which was responsible for major air pollution (Ebinghaus et al. 1999). A similar concentration was detected in the Gulf of Riga compared to the Gdansk Basin (100 ng/g) (Leivuori et al. 2000). Although concentration of mercury in the dumpsite area is not high, a significant increase of Hg in individual stations was detected. So, it is probable that dumped ammunition has an important impact on the level of concentration of mercury in Baltic Sea sediments. Individual cases differences may be related to the degree of ammunition covers corrosion and presence/absence of detonators and primers containing mercury fulminate. Complete recognition of this phenomenon would require additional studies. Moreover, we can expect that covers of ammunition will further corrode and because of that, concentration of mercury in these regions may increase.

### Summary

Mercury, as a metal situated on the top of the most dangerous global pollutants, still remains an important problem for the natural environment. The main sources of mercury in the Baltic Sea are overland runoff, remobilization from sediments, atmospheric deposition, and inflow from the North Sea. Despite many natural sources, Hg is introduced to the environment by anthropogenic activity. The ammunition can be a new potential source of Hg in both terrestrial and marine environment because of mercury fulminate content in blasting caps, which were not removed from some of those ammunitions. An increase of concentration of Hg in soil samples was a result of military activities as well. Significantly increased concentration of Hg in area of active gun range compared to the reference station was a result of firearms usage. Despite the fact that military activities in Poland had finished several dozen years ago, higher concentration of Hg in area of inactive training grounds are still noticeable. Concentration of mercury in these zones of former gun ranges (Hel Peninsula area) was higher compared to the references stations.

The input of mercury to the Baltic Sea has decreased in the past two decades (HELCOM 2011). Sediments are the final location for heavy metals deposition bioturbation and bottom currents lead to remobilization of Hg (Zhang et al. 2012). Twenty to fifty percent Hg deposited in sediments returns to the bottom water (Bełdowski et al. 2009). The forms of mercury are different from each other in mobility, the total concentration of Hg does not reflect stability of mercury in sediments (Forster and Wittmann 1981). Absorption of this metal by land plants and benthic organism causes increased probability of mercury poisoning by humans (Bełdowska et al. 2015). A significant increase of concentration of Hg was detected in areas of the Gdansk Deep, Gotland Basin, and Bornholm Basin. The highest increase of mercury concentration in ammunition dumpsites was observed in western part of the Gotland Basin as compared to the reference station (increase about 206 %). This increase of concentration of mercury in marine dumpsites could be a result of content of organic matter or fine grain. However, after taking into account these factors, increase of concentration of mercury still remains on high level in station with CWA. Differences in level of concentration of Hg in ammunition dumpsites were probably caused by degree of corrosion of weapons in question and presence/absence of mercury fulminate primers. An increase of concentration of mercury was not detected in any station located in Vistula Month area. It was likely connected with the fact that dumped ammunition was not found in this area. CWA, which was detected there, could be a result of transporting these compounds by bottom trawling. Moreover, the important fact is that Vistula mouth is an area of transport but not an accumulation of pollutants.
